# Use of Non-Steroidal Anti-Inflammatory Drugs among Participants in a Mountain Ultramarathon Event

**DOI:** 10.3390/sports5010011

**Published:** 2017-01-29

**Authors:** Sonia Martínez, Antoni Aguiló, Carlos Moreno, Leticia Lozano, Pedro Tauler

**Affiliations:** 1 Research Group on Evidence, Lifestyles & Health, Department of Nursing and Physiotherapy, Research Institute on Health Sciences (IUNICS), University of the Balearic Islands, 07122 Palma de Mallorca, Spain; sonia.martinez@uib.es (S.M.); aaguilo@uib.es (A.A.); carlos.moreno@uib.es (C.M.); leticia.lozano@uib.es (L.L.); 2 Research Group on Evidence, Lifestyles & Health, Department of Fundamental Biology and Health Sciences, Research Institute on Health Sciences (IUNICS), University of the Balearic Islands, 07122 Palma de Mallorca, Spain

**Keywords:** ultramarathon, NSAID consumption, pain relief, medical prescription

## Abstract

The aim of this study was to evaluate and compare the prevalence of non-steroidal anti-inflammatory drugs (NSAID) consumption immediately before, during and immediately after three mountain ultra-endurance runs that differed in their course distance. This observational study took place at the Ultra Mallorca Serra de Tramuntana (Mallorca, Spain), an ultra-endurance mountain event with runners participating either in a 112-km (Ultra, *n* = 58), a 67-km (Trail, *n* = 118) or a 44-km (Marathon, *n* = 62) run competition. Participants in the study answered, within an hour after finishing the competition, a questionnaire focused mainly on NSAIDs consumption. Among study participants, 48.3% reported taking NSAIDs at least for one of the time-points considered: before, during and/or immediately after the competition, with more positive responses (having taken medication) found for the Ultra (60.3%) than for the Trail (49.2%) and the Marathon (35.5%). Among consumers, the Ultra participants reported the lowest intake before and the highest during the race, while participants in the Marathon reported similar consumption levels before and during the race. In conclusion, a high prevalence of NSAID consumption was found among athletes participating in an ultra-endurance mountain event. Competition duration seemed to determine both the prevalence and the chronological pattern of NSAID consumption.

## 1. Introduction

Non-steroidal anti-inflammatory drugs (NSAIDs) are commonly utilised in sports medicine because of their known anti-inflammatory, analgesic, antipyretic and antithrombotic effects [1]. For all classes of athletes, NSAIDs are one of the most commonly used medications for the treatment of musculoskeletal pain and inflammation [2]. In addition to injury treatment, NSAIDs are used as a prophylactic treatment for anticipated pain as well as to enhance the recovery of injuries sustained during an athletic event [3,4,5]. It is noteworthy that some studies have reported high percentages of NSAID consumption without medical prescription, as well as a low level of awareness about NSAID side effects [4,6]. In this sense, it has been reported that NSAID use during endurance exercise is associated not only with gastrointestinal problems [7], but also with increased incidence of hyponatremia [8,9] and with altered renal function [9,10].

Ultra-endurance sports are increasing rapidly in popularity, with a recent exponential growth in the number of events and in athlete participation [11]. Among these events, mountain ultramarathon events are characterized by long distances (>42.195 km) and commonly include a large amount of downhill running, leading to high levels of delayed muscle soreness. Consequently, the extreme nature of these events, combined with their rapidly growing participation base, creates the potential for an increase in analgesic prophylactic use [12]. However, it has been reported that prophylactic use of NSAIDs by athletes may negatively impact the musculoskeletal system [5]. Among other negative effects on the musculoskeletal system, NSAIDs’ inhibitory effects on cyclooxygenase (COX) and prostaglandins may inhibit protein synthesis in the muscle, thus preventing training adaptations [13].

Numerous studies have focused on the incidence of NSAID use during athlete endurance training [4,12,14], prior to competition [4,9,14] and, even, during the Olympic Games [15] and Federation Internationale de Football Association (FIFA) tournaments [16,17], with all events showing a high use of NSAIDs. However, only one previous study has reported on NSAID consumption immediately before and during ultra-endurance events [12]. Furthermore, to our knowledge, no study has compared the NSAID consumption levels for three mountain runs that differ in distances. Therefore, the aim of this study was to evaluate and compare the prevalence of NSAID consumption immediately before, during and immediately after three different mountain ultra-endurance runs (44 km, 67 km and 112 km).

## 2. Materials and Methods

This observational study took place at the Ultra Mallorca Serra de Tramuntana in Mallorca, Spain. The Ultra Mallorca Serra de Tramuntana is an ultra-endurance mountain event with runners participating either in a 112-km (Ultra), a 67-km (Trail) or a 44-km (Marathon) competition run.

The Ultra competition included an elevation gain of about 4450 m, starting at about 100 meters above sea level (m.a.s.l.) and finishing at about 47 m.a.s.l. (with the highest point in the race located at about 1155 m.a.s.l. at km 82). The competition started at 00:00 h and the maximum time allowed to finish the race was 24 h.

The route of the Trail competition coincided with the last 67 km of the Ultra, and thus the two finished at the same place. This competition included an elevation gain of about 2521 m, starting at 409 m.a.s.l., and continued until it reached its highest point at 1155 m.a.s.l. (km 37). The competition started at 08:00 on the same day as the Ultra, and the maximum time allowed to finish the race was 16 h.

The route of the Marathon competition included the last 44 km of the Ultra, and had an elevation gain of approximately 1424 m, starting at about 37 m.a.s.l. and included the same highest point described previously (km 19.5). This competition started at 10:00 h and the maximum time allowed to finish the race was 10 h.

All of the runners of the three competitions were previously informed about the study. Participation requirements for these competitions stipulated that athletes had to be at least 18 years of age. Upon arrival at the finish line, athletes that completed the competitions were invited to participate in this study and 238 (211 men and 27 women) volunteered to do so (11.3% of the finishers). Among them, 58 (one woman) were Ultra runners (11.7% of the 496 finishers), 118 (12 women) participated in the Trail (12.5% of the 942 finishers) and 62 (14 women) completed the Marathon (20.1% of the 308 finishers). Written informed consent was obtained from all the participants in the study. The protocol was in accordance with the Declaration of Helsinki for research on humans and was approved by the University of the Balearic Islands Ethics Research Committee. Data is available upon request to the corresponding author.

Participants in the study answered, within an hour after finishing the competition, a questionnaire focused on NSAIDs consumption based on previous studies [4,12]. For sample characterization, the survey contained questions about personal data such as sex, age, and self-reported weight and height as well as about the weekly training load. The competition in which the runner participated in as well as the competition completion time were also recorded. To assess participant NSAID consumption, questions about habitual NSAID consumption (on a daily basis), and the consumption immediately before the race (within the previous 90 min), during the race and immediately after the race were included. Multiple choice questions about the type and dose of NSAID taken (options given: ibuprofen 400 or 600 mg, diclofenac 50 mg, dexketoprofen 12.5 or 25 mg, salicylic acid 500 or 1000 mg, naproxen 500 mg, others); reasons for consumption (options given: pain prevention, pain relief, injury treatment, others); whether the NSAID was taken with a medical prescription or the source of information for taking the drug (options given: medical prescription, own decision, suggestion of teammates, recommendation of the trainer, others) were also included in the questionnaire.

Statistical analysis was carried out using a statistical package for social sciences (IBM SPSS Statistics 21.0 for Windows). Continuous data are expressed as means ± S.D. Categorical data are shown as frequency counts and percentages. The chi-squared test, with Z statistic, was applied to assess differences between groups in categorical variables. One-way ANOVA with the Least Significant Difference (LSD) post-hoc test was used to analyze differences between groups in continuous variables. *p* < 0.05 was considered statistically significant for all tests performed.

## 3. Results

### 3.1. Characteristics of Participants

Table 1 shows the general characteristics of the study participants, as well as the mean time to complete the competition performed. On account of the presence of a higher number of women, both the mean weight and height of the Marathon participants were significantly lower.

### 3.2. NSAID Consumption

Of the 238 participants in the study, 48.3% reported taking NSAIDs at least at one of the time-points considered (before, during and/or immediately after competition) (Table 2) with significant higher values for the Ultra (60.3%) than for the Marathon (35.5%). For NSAIDs consumption during the race, 37.0% of the participants reported taking NSAIDs, with a significantly lower percentage for the Marathon (17.7%) than for the Ultra (51.7%) and the Trail (39.8%) participants. Only six participants (2.5%) reported taking NSAIDs on a daily basis.

Among consumers, 53.9% reported consuming NSAIDs only during competition and 15.6% only before the competition (Table 3). Ultra participants reported the lowest intake for only before the race and the highest only during the race (5.7% and 65.7% respectively). Marathon participants reported similar consumption values before and during the race (40.9% and 36.4% respectively). Furthermore, the highest proportion of consumers taking NSAIDs only before the race was found among Marathon runners (40.9% vs. 12.1% and 5.7% in the Trail and in the Ultra respectively).

Around 87% of the consumers reported using ibuprofen for all of the consumption times studied (Table 4). While marathon participants reported taking only ibuprofen, Ultra and Trail participants also reported consuming other NSAIDs although with a much lower prevalence. Diclofenac was consumed by one participant before the Ultra, one participant before the Trail and four participants during the Trail; dexketoprofen was consumed by one participant before the Trail, one participant during the Ultra, three participants during the Trail and one participant after the Trail salicylic acid was consumed by one participant after the Trail; naproxen was consumed by one participant before the Trail and two participants during the Trail; and oxicam or piroxicam were consumed by one participant before the Trail and one participant during the Trail.

Table 5 shows ibuprofen consumption during competitions. The consumption during the Ultra was significantly higher than during the Trail (*p* = 0.002) and the Marathon (*p* < 0.001). When this consumption was expressed considering the duration of the competitions, consumption during the Marathon was significantly higher than during the Ultra (*p* = 0.04) and the Trail (*p* = 0.016).

### 3.3. Reasons for NSAID Consumption and Prescription

Reasons for taking NSAIDs cited by the athletes were recorded without considering the time-point for when the athlete had taken the drug. Reasons given by participants were pain prevention (56.4%), pain relief (30.9%), injury treatment (9.1%) and headache (3.6%). Furthermore, among consuming participants, 38.5% reported taking NSAIDs following medical prescription, 30.8% following their own decision, 25.6% following suggestions of teammates and 5.1% following the recommendation of the trainer.

## 4. Discussion

The purpose of the present study was to evaluate and compare the prevalence of NSAID consumption immediately before, during and immediately after three different mountain ultra-endurance runs. The main finding of the present study is the high prevalence of consumption of NSAIDs during the competitions, especially during the Ultra run. It is also noteworthy that while consumption among Ultra and Trail participants was higher during rather than immediately before the competition, consumption among marathon participants was similar immediately before and during the run.

### 4.1. Prevalence of NSAID Consumption

High levels of NSAID use during ultramarathons have been previously reported. Nieman et al. [18] reported a prevalence of NSAID consumption of 72% among a group of 60 participants during a 160-km race. Furthermore, a study of 500 runners in two major North American ultramarathons (161 km) showed that 60.5% of race finishers used NSAIDs during the race [11]. This prevalence is higher than the one found in the present study in the 112-km race, but the difference could be related to the longer duration of the 160-km races. For marathon race consumption, it has been reported that 26% of participants in the 2011 Empire State Marathon consumed NSAIDs during the race [12]. These results are slightly higher than the ones found in the present study for the 44-km run, even though the Empire State Marathon was not a mountain marathon and also included participants in a full-marathon and a half-marathon. The lower prevalence of NSAID consumption during the race found in the present study could be related to the fact that the consumption immediately before the start of the race was not considered as “during the race consumption”, while other studies also did not clarify whether the consumption immediately before the race was considered together with the consumption during the race or is not considered as such [12]. Thus, we consider that the inclusion of this “immediately before” time point, which has not been commonly considered, supposes a strength of the present study.

### 4.2. Effect of Race Duration on the Prevalence and the Pattern of NSAID Consumption

As it has been previously suggested [12], the increasing prevalence in NSAID consumption associated with the increment in competition distance found in the present study could be explained by the increase in the duration of the events, which increases the probability that an athlete may experience pain and turn to NSAIDs for relief. However, in the present study, differences in the pattern of NSAID use for the longest and the shortest run distances were also found. Among Ultra participants, consumption during competition was the preferred choice, while among Marathon participants, consumption before and during the race was very similar, with intermediate figures for the Trail participants. In fact, the highest proportion of NSAID consumers participating in the Ultra as well as the Trail reported consuming NSAIDs only during the race. However, among Marathon consumers, a very similar number of participants reported consuming only before and only during the race. These differences could also be related to the duration of the competition because, even when a common dosage is followed (based on ibuprofen dosage), the mean duration of the Ultra permits participants to take 2–3 NSAID doses during the race, while the shorter Marathon duration limits the consumption to one dose. A much higher consumption during the competition, rather than consumption before the competition, was also found in an Ironman Triathlon where most of the participants took 10–15 h to complete the competition [4], a duration which is between the times of the Ultra and the Trail. However, when the amount of ibuprofen consumed during the races was analyzed, taking into consideration the duration of the races, NSAID consumption during the Marathon was higher than during the Ultra. In fact, the total amount of ibuprofen consumed during the Marathon would correspond to a daily intake of about 2400 mg, which supposes one of the highest medical prescription dosage (i.e., 600 mg, four times daily), and is much higher than the over-the-counter common dosage (1200 mg daily). Therefore, it is possible that factors such as the training level, which is expected to be higher among Ultra participants (reflected, at least in part, by the longer weekly training load reported, as is shown in Table 1), or the intensity, could also influence the NSAID consumption and explain some of the differences found between the longest and the shortest races.

A high NSAID consumption during or immediately before the race may put participants at an elevated risk of adverse events. Two major risks of NSAID use during an ultra-endurance race are the development of renal complications and exercise-associated hyponatraemia. NSAIDs reduce prostaglandin synthesis in the kidney, decreasing the glomerular filtration rate and afferent renal blood flow, which may already be lowered from exercise by as much as 50% [7,14,19]. This combination can impair the athlete’s free water clearance, increasing the risk of developing renal disease and/or hyponatraemia. Furthermore, it has been suggested that NSAIDs use during or after exercise could lead to an impaired muscular recovery from damaging exercise, as well as an inhibition of the skeletal muscle adaptation to training. COX and prostaglandins are important mediators in the responsiveness and subsequent adaptation of connective tissues to mechanical stimuli [13]. Therefore, athletes who take NSAIDs, which inhibit COX isoenzymes and prostaglandins, before, during and even immediately after exercise, could have reduced tissue adaptation to exercise. It has been suggested that NSAIDs taken before and during activity may mask musculoskeletal pain and thus cause athletes to inadvertently allow pathology to progress [5]. Furthermore, ibuprofen, as well as other NSAIDs, has the potential to reduce the rate of matrix production and subsequent tissue repair following microscopic or macroscopic injury [13]. In addition, and in spite of the role of NSAIDs in such problems not yet being clear, NSAIDs may also cause potentially dangerous side-effects in the gastrointestinal tract [7]. In fact, at least two studies have reported higher incidence of gastrointestinal related problems in ultra-endurance athletes taking NSAIDs [4,20]. 

Several authors have reported no beneficial effect of ibuprofen in alleviating muscle soreness and damage after contraction-induced muscle injury [18,21,22,23]. However, and in spite of this observation about ibuprofen, Nieman et al. [18] found that among NSAIDs users during a 160-km race, ibuprofen was used by 86% of the NSAID consumers. These results are very similar to the ones found in the present study, as ibuprofen consumption during the three races made up 87% of the NSAIDs consumed by the whole group of participants, with similar figures before these races. It is noteworthy that diclofenac, which in the present study was used by only two participants, was reported to slightly reduce muscle soreness in spite of the fact that muscle damage was not affected [24].

### 4.3. Reasons for NSAID Consumption and Prescription

In agreement with previous results [4], the main reason given for NSAID use in the present study was pain prevention. Similar results were found when the reasons for taking NSAIDs during the training period and also before and during an Ironman Triathlon competition were analyzed [4]. The findings of the present study are in agreement with the suggestion that participants in mountain ultramarathons consume NSAIDs to prevent delayed-onset muscle soreness because mountain runs involve a great amount of hill descent, which is characterized by predominant eccentric muscle contraction [25]. In fact, the prevalence of NSAID consumption found in ultra-endurance events involving less eccentric exercise was lower than the levels found for the longest race of the present study, as self-reported usage ranging from 30% to 47% was recorded for two Ironman ultradistance triathlons [9,12]. However, it is noteworthy that athletes took NSAIDs mainly without medical prescription. In fact, the percentage of NSAID consumption without medical prescription in the present study (61.5%) was slightly higher than values previously reported [4,6]. These results regarding the amount of participant auto-medication, together with the limited awareness of the side effects [4,6], indicate that participants could result in taking more NSAIDs than the recommended dose, which supposes an additional increased risk factor regarding the related side effects [12]. As has been indicated previously [4], this practice is contrary to the suggestion that, even though the use of NSAIDs is permitted by the World Antidoping Agency, athletes and their physicians should not administer them without medical necessity [26]. The recommendation for considering paracetamol, as well as acetaminophen, as the first-line treatment for musculoskeletal pain due to comparable analgesic efficacy with NSAIDs, but a lower side-effect profile [1,12] should also be reconsidered by athletes and their physicians.

### 4.4. Study Limitations

Some limitations of this study should be acknowledged. The questionnaire used was not formally validated. The relation between consumption of NSAIDs and parameters such as performance or incidence of NSAIDs’ side effects was not explored. Furthermore, being a cross-sectional, observational study, no causal relationship can be drawn between NSAIDs intake and the distance of the run. The very small number of women participants could be considered as a limitation. Finally, using questionnaires could lead to an underestimation of NSAID consumption, which is a common limitation when participants are asked about unhealthy or negative behaviors.

## 5. Conclusions

To the best of our knowledge, this is the first study analyzing and comparing NSAID consumption in three different mountain ultramarathons of increasing distance performed within the same event. A high prevalence of NSAID consumption was found among athletes participating in an ultra-endurance mountain event, most of them without a medical prescription. The duration of the competition seems to determine both the prevalence and the chronological pattern of NSAID consumption; the prevalence of consumption decreased with decreasing duration and higher consumptions during the longest races, although similar consumptions that took place immediately before and during the shortest race were observed.

## Figures and Tables

**Table 1:** General characteristics of participants.

	All Participants (*n* = 238)	Ultra (*n* = 58)	Trail (*n* = 118)	Marathon (*n* = 62)	*p* Value
**Age (years)**	35.7 ± 7.9	35.8 ± 6.5	35.2 ± 8.4	36.6 ±8.0	0.516
**Weight (kg)**	70.6 ± 9.3	71.9 ± 6.3	71.4 ± 9.9	67.9 ± 9.9	0.026
**Height (cm)**	175.1 ± 7.0	177.6 ± 5.1	175.2 ± 7.1	172.5 ± 7.6	<0.001
**Training load (h/week)**	8.26 ± 4.20	10.2 ± 4.0	8.1 ± 4.2	6.8 ± 3.5	<0.001
**Race time (hours)**		17.5 ± 2.8	10.4 ± 1.7	6.2 ± 1.2	

Note: The values are the mean ± S.D. *p* < 0.05 was considered statistically significant (one-way ANOVA).

**Table 2:** Number of non-steroidal anti-inflammatory drugs consumers among participants in the study.

	All Participants (*n* = 238)	Ultra (*n* = 58)	Trail (*n* = 118)	Marathon (*n* = 62)	*p* Value
**Habitual** (*n*, %)	6 (2.5%)	1 (1.7%)	2 (1.7%)	3 (4.8%)	0.501
**Competition Day** (*n*, %)	115 (48.3%)	35 (60.3%)	58 (49.2%)	22 (35.5%) ^&^	0.024
**Before Competition** (*n*, %)	38 (16.0%)	6 (10.3%)	21 (17.8%)	11 (17.7%)	0.405
**During Competition** (*n*, %)	88 (37.0%)	30 (51.7%)	47 (39.8%)	11 (17.7%) ^&,^*	<0.001
**After Competition** (*n*, %)	17 (7.1%)	6 (10.3%)	7 (5.9%)	4 (6.5%)	0.548

Notes: Values are expressed as *n* (% of participants taking non-steroidal anti-inflammatory drugs with respect to number of participants in the study). Habitual indicates daily use of NSAIDs. Competition day indicates consumption either immediately before, during or immediately after the race. ^&^ indicates significant differences between Ultra and Marathon; * indicates significant differences between Trail and Marathon (Z-test). *p* < 0.05 was considered statistically significant (Chi square test).

**Table 3:** Distribution of non-steroidal anti-inflammatory drugs consumers depending on the time-point of consumption.

	All Consumers (*n* = 115)	Ultra (*n* = 35)	Trail (*n* = 58)	Marathon (*n* = 22)	*p* Value
**Only before competition** (*n*, %)	18 (15.6%)	2 (5.7%)	7 (12.1%)	9 (40.9%) ^&,^*	0.046
**Only during competition** (*n*, %)	62 (53.9%)	23 (65.7%)	31(53.4%)	8 (36.4%) ^&^	0.004
**Only after competition** (*n*, %)	8 (7.0%)	3 (8.6%)	3 (5.2%)	2 (9.1%)	0.659
**Before and during competition** (*n*, %)	18 (15.6%)	4 (11.4%)	13 (22.4%)	1 (4.5%)	0.075
**Before and after competition** (*n*, %)	1 (0.9%)	0	1 (1.7%)	0	0.600
**During and after competition** (*n*, %)	7 (6.1%)	3 (8.6%)	3 (5.2%)	1 (4.5%)	0.482
**Before, during and after competition** (*n*, %)	1 (0.9%)	0	0	1 (4.5%)	0.240

Notes: Values are expressed as *n* (% with respect to number of consumers). *p* < 0.05 was considered statistically significant (Chi square test). ^&^ indicates significant differences between Ultra and Marathon; * indicates significant differences between Trail and Marathon (Z-test).

**Table 4:** Number of consumers taking ibuprofen or another non-steroidal anti-inflammatory drug.

		All Consumers (*n* = 115)	Ultra (*n* = 35)	Trail (*n* = 58)	Marathon (*n* = 22)
**Before competition**	Ibuprofen (*n*, %)	33 (86.8%)	5 (83.3%)	17 (81.0%)	11 (100%)
	Others (*n*, %)	5 (13.2%)	1 (16.7%)	4 (19.0%)	0
**During competition**	Ibuprofen (*n*, %)	77 (87.5%)	28 (93.3%)	37 (78.7%)	11 (100%)
	Others (*n*, %)	11 (12.5%)	2 (6.7%)	10 (21.3%)	0
**After competition**	Ibuprofen (*n*, %)	15 (88.2%)	6 (100%)	5 (71.4%)	4 (100%)
	Others (*n*, %)	2 (11.8%)	0	2 (28.6%)	0

Notes: Values are expressed as *n* (% with respect to number of consumers). Others include diclofenac, dexketoprofen, salicylic acid, naproxen and oxicam or piroxicam.

**Table 5:** Ibuprofen consumption during competitions.

	All Consumers (*n* = 62)	Ultra (*n* = 23)	Trail (*n* = 30)	Marathon (*n* = 9)	*p* Value
**mg**	835.5 ± 400.9	1052 ± 456	713 ± 206 ^#^	689 ± 163 ^&^	0.03
**mg/hour competition**	70.3 ± 31.2	61.3 ± 32.9	68.9 ± 27.0	96.7 ± 27.4 ^&,^*	0.013

Notes: The values are the mean ± S.D. *p* < 0.05 was considered statistically significant (one-way ANOVA). ^#^ indicates significant differences between Ultra and Trail; ^&^ indicates significant differences between Ultra and Marathon; * indicates significant differences between Trail and Marathon.
